# Reply to the comment on "the association between cognitive function trajectories and all-cause mortality in Middle-aged and older Chinese adults with cardiovascular disease: A longitudinal study from CHARLS"^☆^

**DOI:** 10.1016/j.ijcrp.2026.200645

**Published:** 2026-04-22

**Authors:** Xiaopeng Song, Yufei Wang, Hua Chen

**Affiliations:** a Department of Cardiology, Inner Mongolia Autonomous Region People's Hospital, Hohhot City, People's Republic of China; b Graduate School, Inner Mongolia Medical University, Hohhot City, People's Republic of China

Dear Editor,

We thank Peddapalegani and colleagues [1] for their thoughtful comments on our recent study of cognitive trajectories and all-cause mortality in patients with cardiovascular disease [2].

First, we agree that deriving trajectories from only three waves may limit the distinction between sustained decline and short-term fluctuation. However, extending trajectory derivation to the 2018 and 2020 CHARLS waves is methodologically challenging because the cognitive assessment changed after 2015, particularly for word recall, affecting comparability across waves. Recent work has shown that harmonization or score equating is required before later-wave data can be validly combined with earlier assessments [3]. We therefore restricted trajectory identification to 2011-2015 and assessed robustness using alternative clustering approaches, internal validity indices, and sensitivity analyses with k = 2, 3, and 4. These results supported the overall robustness of the identified patterns.

Second, we agree that a global cognitive composite may obscure heterogeneity across domains. We therefore examined subdomain-specific rates of change, as described in Sections 3.3 and 4.3 of the original article. However, formal domain-specific trajectory modeling remains limited. Such analyses are particularly challenging for visuospatial function in CHARLS, as this domain is assessed using a single binary item.

Third, the stronger association observed among participants with hypertension is consistent with shared vascular mechanisms, but our data cannot determine whether adverse cognitive trajectories reflect cumulative vascular burden or an independent prognostic signal.

## CRediT authorship contribution statement

**Xiaopeng Song:** Conceptualization, Formal analysis, Writing – original draft. **Yufei Wang:** Data curation, Methodology, Writing – original draft. **Hua Chen:** Conceptualization, Investigation, Project administration, Supervision, Writing – review & editing.

## Funding

This work was supported by Inner Mongolia 10.13039/501100000691Academy of Medical Sciences, 10.13039/100018696Health Committee of Inner Mongolia Autonomous Region, China (grant number 2023GLLH0010).

## Declaration of interest

All authors confirm that this manuscript is original, has not been published elsewhere, and is not under consideration by another journal. All authors have approved the final version of the manuscript and agree with its submission to *International Journal of Cardiology Cardiovascular Risk and Prevention*. The authors declare no competing interests.
